# Experience with Pediatric Chronic Immune Thrombocytopenia over 30 Years in the Era before Eltrombopag

**DOI:** 10.3390/children11091051

**Published:** 2024-08-28

**Authors:** Begum S. Koc, Gul Nihal Ozdemir, Javid Alakbarli, Hilmi Apak, Tiraje Celkan

**Affiliations:** 1 Department of Pediatric Hematology and Oncology, Cerrahpasa Medical Faculty, Istanbul University, Istanbul 34696, Turkey; 2 Department of Pediatric Hematology and Oncology, Istinye University, Istanbul 34320, Turkey

**Keywords:** children, thrombocytopenia, chronic ITP, splenectomy

## Abstract

Background: There is limited information on the natural course of chronic ITP in children. We aimed to evaluate the clinical and demographic characteristics of children with chronic ITP in the era before the availability of eltrombopag. Methods: A total of 86 children with chronic ITP between 1978–2014 were included. Demographic findings, laboratory results, clinical signs, bleeding scores, response time and time of complete remission were recorded. Results: The male/female ratio was 1.09, and median follow-up time was 3 years (range: 1.5–17 years). The median age at diagnosis of chronic ITP was 7 years (range: 2–17), and the median initial platelet count was 10 × 10^9^/L (range: 1–66 × 10^9^/L). Petechiae/ecchymoses were the most common clinical sign (86%) and followed by mucosal bleeding (39.5%). Severe bleeding was seen in 5% of the patients. None of them had intracranial hemorrhage. Twenty patients underwent splenectomy, and the rate of complete remission was 70%. Spontaneous complete remission was seen in 29% of the patients, and the median time to spontaneous complete remission was 3 years. Conclusions: Our study showed that almost one-third of patients with chronic ITP experienced spontaneous complete remission in an average of 3 years, and splenectomy provided satisfactory results in severe cases. This study demonstrates the natural history of chronic ITP in childhood before the era of eltrombopag.

## 1. Introduction

Immune thrombocytopenia (ITP) is a common and usually self-limiting acquired autoimmune bleeding disorder that presents with low platelet counts in childhood [1,2]. The definition, terminology and outcome criteria of ITP were revised by the International Working Group (IWG) in 2009 [3]. The first 3-month period after diagnosis was defined as “newly diagnosed”, the period between 3–12 months as “persistent”, and ITP lasting more than 12 months as “chronic”. Approximately 70% of patients with acute ITP have ‘complete remission’ within six months, 40% of children with ITP have a persistent course (3–12 months), and 10–20% become chronic (>12 months) [4,5,6]. Adolescent age group, insidious onset, mild bleeding symptoms and high platelet count at diagnosis are shown as risk factors for chronicity [7,8].

Therapeutic choices are complex in patients with chronic ITP, and the primary goal is to improve health-related quality of life (HRQoL) and control bleeding rather than increasing platelet counts. Corticosteroids and intravenous immunoglobulins (IVIG) are used as first-line treatments in case of bleeding symptoms or increased bleeding risk [9,10]. Second-line therapeutic options include, more recently, thrombopoietin receptor agonists (TPO-RAs) and immunosuppressive drugs such as rituximab or splenectomy. Thrombopoietin-receptor agonists (TPO-RAs), eltrombopag, and romiplostim are safe and effective with excellent response rates (~80%) for children with chronic ITP [11,12,13,14]. However, it appears that these drugs are used for a long time for sustained response in chronic ITP, and the long-term toxicity of these new drugs in children with ITP is not well-known. We suggest that we need more information about the natural course of chronic ITP in children.

There is limited information on the natural course of chronic ITP in children in the literature. Here, we aimed to evaluate the clinical and demographic characteristics, response to treatment agents, and spontaneous remission of children with chronic ITP in the period before the use of TPO-RAs.

## 2. Materials and Methods

The data of children with ITP diagnosed between 1978 and 2014 at Cerrahpasa University Hospital were reviewed. This is one of the biggest tertiary hospitals in Istanbul and in Turkey with more than 1000 admissions of children with hematological diseases per year.

Patients with chronic ITP, which was redefined according to the revised IWG criteria, were included in the study. Platelet counts of less than 100 × 10^9^/L and persistence for more than 12 months following the initial diagnosis were defined as chronic ITP. Complete response was defined as the platelet count reached 100 × 10^9^/L. Complete remission was defined as platelet count > 100 × 10^9^/L for >3 months. Spontaneous complete remission was defined as platelet count > 100 × 10^9^/L for >3 months without splenectomy and medical treatment. The bleeding assessment tool (ISTH-BAT) was used to objectively evaluate bleeding symptoms. According to the ISTH-BAT scoring system, ≥3 points necessitate the requirement for medical/surgical hemostasis and/or blood transfusion to stop bleeding and the severity of bleeding, <3 points means that bleeding is not clinically significant [15].

Our approach in the management of chronic ITP is “wait-and-see” for patients with a platelet count of >10 × 10^9^/L and/or no bleeding. Treatment, if needed, includes IVIG, corticosteroids, and anti-D. The dosage schemes used in our clinic are as follows: IVIG (0.8–1 g/kg/day, 1 or 2 days) or high-dose steroid (30 mg/kg/day methylprednisolone, 3 days) or low-dose steroid (2 mg/kg/day methylprednisolone per oral for 14 days, tapered and stopped in the 3rd week). Anti-D treatment is an option for Rh (+) cases without splenectomy (50–70 μg/kg/day single dose or 20 μg/kg/day for 3 days). Bone marrow examination is performed if clinically indicated or before corticosteroid therapy. Second-line treatment alternatives, such as rituximab, dapsone, danazole, and vincristine, have been used for chronic ITP. At the time of this study, TPO-RAs were not yet available for use in Turkey. Indications for splenectomy are persistent mucosal bleeding that does not respond to drug therapy and severe bleeding attacks leading to repeated hospitalizations.

### Statistical Analysis

Data were analyzed using the SPSS for Windows version 13.0 (SPSS, Chicago, IL, USA). Subgroups of patients were characterized and compared using descriptive statistics. The differences in dichotomous variables were analyzed by Pearson’s χ2 test, and the differences in continuous variables were analyzed by analysis of variance (ANOVA), Man Whitney U test, Chi-Square test and independent *t*-test. *p*-value of <0.05 was considered statistically significant.

## 3. Results

Between 1978 and 2014, a total of 564 children were diagnosed with ITP in our clinic; on follow-up, 86 (41 girls and 45 boys) developed chronic ITP. The rate of chronicity was found to be 15.2% among all children diagnosed with ITP. The median age at diagnosis of chronic ITP was 7 years (range: 2–17 years). Twenty-four patients (28%) were older than 10 years of age, and most of them (n:57, 66%) were older than 6 years old. The male/female ratio was 1.09. The median initial platelet count was 10 × 10^9^/L at the time of newly diagnosed ITP (range: 1–66 × 10^9^/L). Chronic ITP patients were grouped according to initial platelet counts at the time of newly diagnosed ITP in three groups: platelet count < 20 × 10^9^/L (n:54), 20–50 × 10^9^/L (n:24), and >50–100 × 10^9^/L (n:2). The initial platelet count data of six patients could not be obtained. The most commonly used agents for bleeding control at the time of newly diagnosed ITP were corticosteroids (n:52) and IVIG (n:25). Only one patient received anti-D Ig. Eight patients did not receive any treatment and were followed up by “wait and see”.

The median follow-up time of patients with chronic ITP was 3 years (range: 1–17 years). The most common clinical bleeding symptoms on follow-up were petechiae and/or ecchymoses (n:72), epistaxis (n:21) and gum bleeding (n:13). Hematuria (n:3), gastrointestinal bleeding (n:1), subconjunctival hemorrhage (n:1), and menorrhage (n:1) were also observed as more severe bleeding episodes. None of the patients with chronic ITP had intracranial hemorrhage. Most of the patients (n:73, 84.8%) had an ISTH-BAT score of <3, and 15.2% had a score of ≥3 during follow-up. Demographic and clinical characteristics of patients are shown in Table 1 and Table 2.

Viral serology results were obtained from 35 patients; HBsAg (n:1), parvovirus IgM (n:1) and EBV DNA (n:1) were positive in three patients. One patient also had a history of *Helicobacter pylori* treatment. Anti-nuclear antibody (ANA) test was performed in 27 patients, and positivity was detected in 8; an anti-dsDNA test was performed in 20, and positivity was detected in 3; direct Coombs test was investigated in 41 patients and positivity was detected in 8. Four patients were diagnosed with SLE on follow-up. One patient was diagnosed with Evans syndrome.

On follow-up, patients, when indicated, were treated either by corticosteroids or IVIG according to clinical decision. Our observation with these drugs was that the majority of patients had a good response to IVIG and steroids, but the response was transient in chronic ITP. Second-line treatments that were used rarely in selected patients were rituximab (n:5), dapsone (n:3), danazol (n:2) and vincristine (n:1). A transient partial response was only observed to dapsone treatment; there was no complete or partial response to the other agents (rituximab, danazol, vincristine). 

Splenectomy was performed in twenty patients (23%). The median age for splenectomy was 10 years (range: 5.5–16 years). The median time of splenectomy after the diagnosis was 2 years (range: 6 months–14 years). Complete response was observed in 16 patients (80%) after splenectomy, and partial response (platelet count: 50–100 × 10^9^/L) was observed in two (10%). No response was obtained in two patients (10%). Two of the complete responding patients later relapsed after 3 years from splenectomy. Complete remission was achieved in 14 patients (70%) after splenectomy.

**Figure 1:**
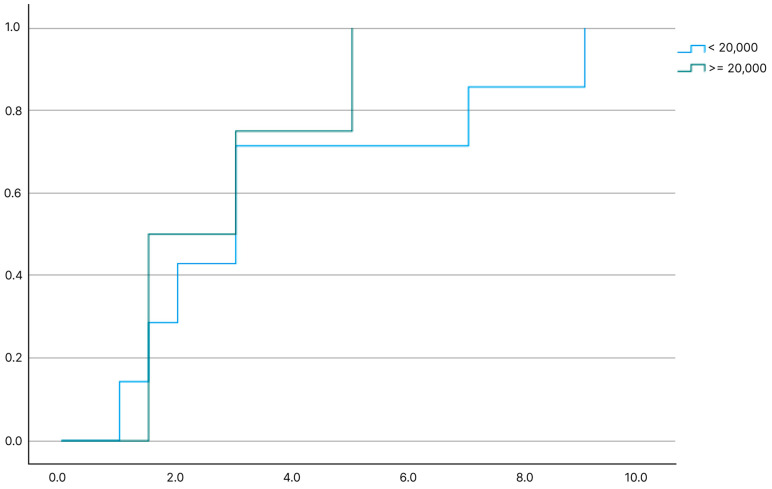
The probability of remission in patients with chronic ITP according to the platelet count at initial diagnosis of ITP.

**Figure 2:**
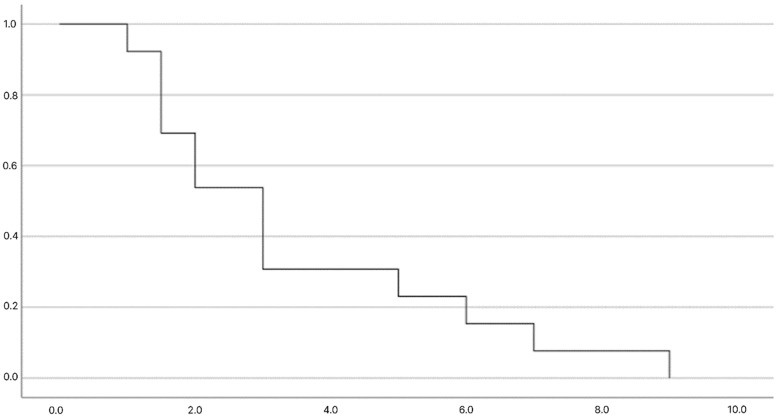
Recovery of the patients with chronic ITP over time.

Overall complete remission rate was 45% (n:39); 14 of them underwent splenectomy, and the remaining 29% (n:25) had spontaneous remission. The rate of spontaneous complete remission was 29% in all patients with chronic ITP, and the median time to spontaneous complete remission was 3 years (range: 1–9 years). Patients with spontaneous complete remission (n:25) were compared with patients without complete remission (n:47). There was no statistically significant difference between the two groups in terms of age, gender, and the time of complete response to initial treatment, initial platelet counts and the initial treatment agent (corticosteroid or IVIG) at the time of newly diagnosed ITP (*p* > 0.5) (Table 3). There was no statistically significant difference between recovery times according to platelet groups (*p* = 0.52). The probability of remission in patients with chronic ITP according to the platelet count at initial diagnosis of ITP was shown in Figure 1, and recovery of the patients with chronic ITP by the time is shown in Figure 2.

## 4. Discussion

After the new definition by IWG, the rate of chronic ITP has been reported between 10 to 20% [6,16]. The rate of chronicity was found to be lower at 15.2% in our study than in the study of Chotsampancharoen et al., which was found to be 27.9% [17]. We previously reported that the male/female ratio was 1.1 in children with newly diagnosed ITP, and in this study male/female ratio was also similarly 1.09 in children with chronic ITP [18]. Kühne et al. also reported a similar ratio of male/female in acute and chronic forms in the study of 2450 children with ITP [19]. In the literature, many studies have reported that older children are more likely to have chronic ITP [2,5,8]. In our cohort, most of the children (66%) with chronic ITP were older than 6 years of age.

A study from the UK reported that only 5% (or less) of children with ITP experience severe bleeding, with the most common severe bleeding sites nose and gastrointestinal tract [20]. In a systematic review including 118 studies with data from 10.908 patients, the rate of intracranial bleeding in chronic ITP was 1.6% [21]. In our study, all of the patients with severe bleeding had initial platelet count below <20 × 10^9^/L, and sites of severe bleeding were the gastrointestinal tract, nose, urogenital tract, subconjunctiva and gum. None of our patients had intracranial hemorrhage. Most of the patients had mild bleeding symptoms. A bleeding scoring system has been developed for patients with ITP, but it is not commonly used in clinical practice [22]. Hematologists are used to ISTH-BAT (bleeding assessment tool) in bleeding disorders. However, ISTH-BAT was used to predict future bleeding in hereditary thrombocytopenias and platelet function disorders in the study by Gresele et al. [23]. We also used this scoring system considering all bleeding events of the patients with chronic ITP during follow-up. To our knowledge, this is the first study in which ISTH-BAT was used to assess the severity of bleeding events in patients with chronic ITP. According to the ISTH-BAT scoring system, most patients (84.7%) with chronic ITP had <3 points, which means that their bleeding events were not clinically significant. There were only two patients (2.3%) who had high scores (6 points), and these patients’ initial platelet counts were <5 × 10^9^/L.

The prognostic significance of the choice of initial therapy in newly diagnosed ITP in the development of chronicity is controversial. Additionally, we compared data of the patients with acute ITP [18] published in 2015 by our center with the data of the same patients who were in our study in terms of the first treatment agent effect. Patients diagnosed with acute ITP between 1976 and 2010 were included in that study, and six patients diagnosed with chronic ITP between 2011 and 2015 were excluded before comparison. Among patients with acute ITP, IVIG was administered to 134 patients as the first treatment and 15.7% (n:21) of them were chronic during follow-up. In patients with acute ITP who received steroids in the first treatment (n:235), 21% (n:50) of those became chronic during follow-up. The rate of chronicity was found to be higher in the patients who received steroids as first-line treatment in newly diagnosed ITP.

The effect of the first treatment on remission is also a matter of debate. In a similarly long-term (more than 38 years) study involving 113 children, Chotsampancharoen et al. reported that initial platelet counts and the initial treatment agent used were shown to be the only predictive factors for remission and the rate of remission was higher in patients with initial platelet count > 60 × 10^9^/L and in patients using steroids in first-line therapy [17]. Jayabose et al. reported that age, gender, initial platelet count, first-line therapy, and response time to first-line therapy do not have any prognostic significance on spontaneous remission in children with chronic ITP [24]. Tamary et al. [25] reported that neither age nor platelet count at the time of newly diagnosed ITP had any effect on remission. In our study, we could not find any relationship between the patients with and without spontaneous remission in terms of initial platelet count, first-line therapy and response time to first-line therapy in chronic ITP.

In our study, the rate of splenectomy was 23%, and the rate of complete remission after splenectomy was 70%. According to the literature, splenectomy rates ranged from 6 to 40% [26,27,28,29]. In the study by Donato et al., splenectomy was performed in 13% of children with chronic ITP, and the rate of achieving remission was 73% [30]. Bansal et al. reported that they performed splenectomy in 18 of 270 (6.7%) cases, and the rate of achieving remission was 50% [31]. George et al. reviewed case series of splenectomized children with refractory ITP and found that two-thirds of patients achieved durable complete remission after splenectomy [32]. In recent years, it has been reported that splenectomy is performed with decreasing frequency in case series. The rates of splenectomy ranged from 10 to 40% depending on the period of study, and the remission rates after splenectomy ranged from 40 to 75%. [26,27,28,29]. Despite the reported success rates, overall splenectomy rates consistently declined in children with ITP between 2005 and 2014 [26]. Splenectomy is less preferred today because of the long-term risk of complications and the availability of second-line treatment agents such as TPO-RAs (eltrombopag and romiplostim). When there is no other option for medical treatment, satisfactory results can be achieved in severe cases with splenectomy.

In our study, the rate of spontaneous complete remission was 29% in non-splenectomized children with chronic ITP. Schifferli et al. [5] have shown that 28% of children with chronic ITP had spontaneous remission in 2-year follow-up. Donato et al. reported that spontaneous remission was achieved in 26.5% of non-splenectomized patients with chronic ITP up to 10 years after first diagnosis [30]. According to the results of studies by Jayabose et al. [24] and Bansal et al. [31], spontaneous remission was achieved in approximately 3–4 years in children with chronic ITP. In our study, the median time for spontaneous complete remission was found to be 3 years in the patients with chronic ITP; this result was in accordance with the literature. “The Childhood ITP Recovery Score” has been developed with an online calculation system for chronic type risk estimation. However, a robust scoring system that can predict chronic ITP in the management of ITP has not yet been found [6].

Current guidelines recommend careful waiting rather than treatment with corticosteroids or intravenous immunoglobulin (IVIG) in newly diagnosed children with no bleeding or only minor bleeding. Corticosteroids or immunoglobulins are recommended as first-line therapy for the treatment of newly diagnosed ITP in children with non-life-threatening bleeding. Second-line therapeutic options are recommended for patients who fail to receive remission [33]. Second-line treatment options in children include rituximab and splenectomy, as well as thrombopoietin (TPO) receptor agonists (TPO-RAs) such as romiplostim and eltrombopag. TPO-RAs have been shown to be effective in the treatment of chronic ITP in children and adults [34]. The oral use of TPO-RA-eltrombopag was approved by the US Food and Drug Administration (FDA) for use in children over 1 year of age with chronic ITP in 2015. Given the variable and transient response, frequent relapses, and toxicities associated with corticosteroids and IVIGs, TPO-RAs are good options for pediatric patients with ITP for adverse effects.

### Limitations

This study has a number of limitations. Given that the study population was an observational cohort, treatment decisions were at the discretion of the physician. The severity of bleeding at the time of diagnosis was not recorded in a large number of patients, which was problematic for the final data analysis.

## 5. Conclusions

This study reflects the pre-TPO-RA period and also demonstrates the natural history of chronic ITP, as there was no sustained complete response to any second-line medical treatment over a period of 36 years. We know that some patients with chronic ITP recover spontaneously after a certain period of time in their natural course. Approximately one-third of patients with chronic ITP experienced spontaneous complete remission an average of 3 years after the new diagnosis, and when there is no other option for medical treatment in severe cases, satisfactory results can be achieved with splenectomy. If there is no emergency, we recommend waiting about 3–4 years for spontaneous remission before performing splenectomy in the treatment of chronic ITP.

## Figures and Tables

**Table 1:** Demographic and clinical characteristics of the children with chronic ITP at the time of newly diagnosed ITP.

**Gender**	**n**	**%**
Female	41	47.6
Male	45	52.3
**Age (years)**	**n**	**%**
0–6	29	33.7
6–10	33	38.4
10–17	24	27.9
**Initial platelet counts (×10^6^/L)**	**n**	**%**
<20,000	54	62.8
20,000–50,000	24	27.9
>50,000	2	2.3
**Bleeding symptoms**	**n**	**%**
Skin bleeding	72	83.7
Epistaxis	21	24.4
Gum bleeding	13	15.1
Hematuria	3	3.5
Menorrhage	1	1.2
Gastrointestinal bleeding	1	1.2
Subconjunctival hemorrhage	1	1.2
Intracranial hemorrhage	0	0
**Initial treatment**	**(n)** **78**	**(%)** **90.7**
Corticosteroid	52	60.5
IVIG	25	29
“Wait and see”	8	9.3
Anti-D	1	1.2

**Table 2:** Median values of demographic and clinical characteristics in children with chronic ITP.

	Median Value (Range)
Age at onset of chronic ITP (years)	7 (2–17)
Initial platelet counts (×10^6^/L)	10 (1–66)
Time of complete response (days)	5 (2–33)
Follow-up time (years)	3 (1–17)

**Table 3:** Comparison of the non-splenectomized patients with and without spontaneous complete remission.

	Patients with Spontaneous Complete Remission (n:25)		Patients without Spontaneous Complete Remission (n:41)		
	Mean ± Sd	Median (25–75%)	Mean ± Sd	Median (25–75%)	*p*
Age	7.2 ± 4.0	7 (5–10)	7.1 ± 4.1	7 (4–9)	0.93 ***
Initial platelet count (×10^9^/mm^3^)	19.1 ± 20.8	8 (3–27)	19.0 ± 26.4	12 (3–21)	0.95 *
Complete response time after the initial treatment (days)	52.9 ± 78.3	12 (4–90)	19.0 ± 30.9	3 (2–21)	0.08 *
	n	%	n	%	
Corticosteroid (initial treatment)	19	76	25	61	0.25 **
IVIG (initial treatment)	4	16	16	39	
Girls	11	48	20	49	0.93 **
Boys	13	52	21	51	

* Mann–Whitney U test; ** Chi-Square test; *** Independent *t*-test.

## Data Availability

The data presented in this study will be shared upon reasonable requests from the corresponding author but cannot be shared in public as authors have no permission granted by the ethical board.

## Abbreviations

ANA	Anti-nuclear antibody
BAT	Bleeding assessment tool
ISTH	International Society on Thrombosis and Haemostasis
ITP	Immune thrombocytopenic purpura/Immune thrombocytopenia
IVIG	Intravenous immunoglobulin
IWG	International Working Group
TPO-RA	Thrombopoietin receptor agonist

## References

[1] Despotovic J.M., Grimes A.B. (2018). Pediatric ITP: Is it different from adult ITP?. Hematol. Am. Soc. Hematol. Educ. Program..

[2] Cines D.B., McMillan R. (2007). Pathogenesis of chronic immune thrombocytopenic purpura. Curr. Opin. Hematol..

[3] Rodeghiero F., Stasi R., Gernsheimer T., Michel M., Provan D., Arnold D.M., Bussel J.B., Cines D.B., Chong B.H., Cooper N. (2009). Standardization of terminology, definitions and outcome criteria in immune thrombocytopenic purpura of adults and children: Report from an international working group. Blood.

[4] Kühne T., Imbach P., Bolton-Maggs P.H., Berchtold W., Blanchette V., Buchanan G.R., Intercontinental Childhood ITP Study Group (2001). Newly diagnosed idiopathic thrombocytopenic purpura in childhood: An observational study. Lancet.

[5] Schifferli A., Holbro A., Chitlur M., Coslovsky M., Imbach P., Donato H., Elalfy M., Graciela E., Grainger J., Holzhauer S. (2018). A comparative prospective observational study of children and adults with immune thrombocytopenia: 2-year follow-up. Am. J. Hematol..

[6] Roşu V.E., Spoială E.L., Roşu T.S., Ivanov A.V., Mocanu A., Munteanu A., Lupu V.V., Miron I., Gavrilovici C. (2022). The Use of Clinical Scores in the Management of Immune Thrombocytopenic Purpura in Children. Front. Pediatr..

[7] Schmidt D.E., Wendtland Edslev P., Heitink-Pollé K.M.J., Mertens B., Bruin M.C.A., Kapur R., Vidarsson G., van der Schoot C.E., Porcelijn L., van der Bom J.G. (2021). A clinical prediction score for transient versus persistent childhood immune thrombocytopenia. J. Thromb. Haemost..

[8] Heitink-Pollé K.M., Nijsten J., Boonacker C.W., de Haas M., Bruin M.C. (2014). Clinical and laboratory predictors of chronic immune thrombocytopenia in children: A systematic review and meta-analysis. Blood.

[9] Provan D., Stasi R., Newland A.C., Blanchette V.S., Bolton-Maggs P., Bussel J.B., Chong B.H., Cines D.B., Gernsheimer T.B., Godeau B. (2010). International consensus report on the investigation and management of primary immune thrombocytopenia. Blood.

[10] Bolton-Maggs P. (2003). Severe bleeding in idiopathic thrombocytopenic purpura. J. Pediatr. Hematol. Oncol..

[11] Cooper N., Ghanima W. (2019). Immune Thrombocytopenia. N. Engl. J. Med..

[12] Bussel J.B., de Miguel P.G., Despotovic J.M., Grainger J.D., Sevilla J., Blanchette V.S., Krishnamurti L., Connor P., David M., Boayue K.B. (2015). Eltrombopag for the treatment of children with persistent and chronic immune thrombocytopenia (PETIT): A randomised, multicentre, placebo-controlled study. Lancet Haematol..

[13] Grainger J.D., Locatelli F., Chotsampancharoen T., Donyush E., Pongtanakul B., Komvilaisak P., Sosothikul D., Drelichman G., Sirachainan N., Holzhauer S. (2015). Eltrombopag for children with chronic immune thrombocytopenia (PETIT2): A randomised, multicentre, placebo-controlled trial. Lancet.

[14] Neunert C., Despotovic J., Haley K., Lambert M.P., Nottage K., Shimano K., Bennett C., Klaassen R., Stine K., Thompson A. (2016). Thrombopoietin Receptor Agonist Use in Children: Data from the Pediatric ITP Consortium of North America ICON2 Study. Pediatr. Blood Cancer.

[15] Elbatarny M., Mollah S., Grabell J., Bae S., Deforest M., Tuttle A., Hopman W., Clark D.S., Mauer A.C., Bowman M. (2014). Normal range of bleeding scores for the ISTH-BAT: Adult and pediatric data from the merging project. Haemophilia.

[16] Demircioğlu F., Saygi M., Yilmaz S., Oren H., Irken G. (2009). Clinical features, treatment responses, and outcome of children with idiopathic thrombocytopenic purpura. Pediatr. Hematol. Oncol..

[17] Chotsampancharoen T., Sripornsawan P., Duangchoo S., Wongchanchailert M., McNeil E. (2017). Clinical outcome of childhood chronic immune thrombocytopenia: A 38-year experience from a Single Tertiary Center in Thailand. Pediatr. Blood Cancer.

[18] Yildiz I., Ozdemir N., Celkan T., Soylu S., Karaman S., Canbolat A., Dogru O., Erginoz E., Apak H. (2015). Initial Management of Childhood Acute Immune Thrombocytopenia: Single-Center Experience of 32 Years. Pediatr. Hematol. Oncol..

[19] Kühne T., Buchanan G.R., Zimmerman S., Michaels L.A., Kohan R., Berchtold W., Imbach P., Intercontinental Childhood ITP Study Group (2003). A prospective comparative study of 2540 infants and children with newly diagnosed idiopathic thrombocytopenic purpura (ITP) from the Intercontinental Childhood ITP Study Group. J. Pediatr..

[20] Bolton-Maggs P.H., Moon I. (1997). Assessment of UK practice for management of acute childhood idiopathic thrombocytopenic purpura against published guidelines. Lancet.

[21] Neunert C., Noroozi N., Norman G., Buchanan G.R., Goy J., Nazi I., Kelton J.G., Arnold D.M. (2015). Severe bleeding events in adults and children with primary immune thrombocytopenia: A systematic review. J. Thromb. Haemost..

[22] Rodeghiero F., Michel M., Gernsheimer T., Ruggeri M., Blanchette V., Bussel J.B., Cines D.B., Cooper N., Godeau B., Greinacher A. (2013). Standardization of bleeding assessment in immune thrombocytopenia: Report from the International Working Group. Blood.

[23] Gresele P., Falcinelli E., Bury L., Pecci A., Alessi M.C., Borhany M., Heller P.G., Santoro C., Cid A.R., Orsini S. (2021). The ISTH bleeding assessment tool as predictor of bleeding events in inherited platelet disorders: Communication from the ISTH SSC Subcommittee on Platelet Physiology. J. Thromb. Haemost..

[24] Jayabose S., Levendoglu-Tugal O., Ozkaynkak M.F., Visintainer P., Sandoval C. (2004). Long-term outcome of chronic idiopathic thrombocytopenic purpura in children. J. Pediatr. Hematol. Oncol..

[25] Tamary H., Kaplinsky C., Levy I., Cohen I.J., Yaniv I., Stark B., Goshen Y., Zaizov R. (1994). Chronic childhood idiopathic thrombocytopenia purpura: Long-term follow-up. Acta Paediatr..

[26] Bhatt N.S., Bhatt P., Donda K., Dapaah-Siakwan F., Chaudhari R., Linga V.G., Patel B., Lekshminarayanan A., Bhaskaran S., Zaid-Kaylani S. (2018). Temporal trends of splenectomy in pediatric hospitalizations with immune thrombocytopenia. Pediatr. Blood Cancer.

[27] Rodeghiero F. (2018). A critical appraisal of the evidence for the role of splenectomy in adults and children with ITP. Br. J. Haematol..

[28] Kühne T., Blanchette V., Buchanan G.R., Ramenghi U., Donato H., Tamminga R.Y., Rischewski J., Berchtold W., Imbach P., Intercontinental Childhood ITP Study Group (2007). Splenectomy in children with idiopathic thrombocytopenic purpura: A prospective study of 134 children from the Intercontinental Childhood ITP Study Group. Pediatr. Blood Cancer.

[29] Ahmed R., Devasia A.J., Viswabandya A., Lakshmi K.M., Abraham A., Karl S., Mathai J., Jacob P.M., Abraham D., Srivastava A. (2016). Long-term outcome following splenectomy for chronic and persistent immune thrombocytopenia (ITP) in adults and children: Splenectomy in ITP. Ann. Hematol..

[30] Donato H., Picón A., Rapetti M.C., Rosso A., Schvartzman G., Drozdowski C., Di Santo J.J. (2006). Splenectomy and spontaneous remission in children with chronic idiopathic thrombocytopenic purpura. Pediatr. Blood Cancer.

[31] Bansal D., Bhamare T.A., Trehan A., Ahluwalia J., Varma N., Marwaha R.K. (2010). Outcome of chronic idiopathic thrombocytopenic purpura in children. Pediatr. Blood Cancer.

[32] George J.N. (2006). Management of patients with refractory immune thrombocytopenic purpura. J. Thromb. Haemost..

[33] Russo G., Parodi E., Farruggia P., Notarangelo L.D., Perrotta S., Casale M., Cesaro S., Del Borrello G., Del Vecchio G.C., Giona F. (2024). Recommendations for the management of acute immune thrombocytopenia in children. A Consensus Conference from the Italian Association of Pediatric Hematology and Oncology. Blood Transfus..

[34] Bidika E., Fayyaz H., Salib M., Memon A.N., Gowda A.S., Rallabhandi B., Cancarevic I. (2020). Romiplostim and Eltrombopag in Immune Thrombocytopenia as a Second-Line Treatment. Cureus.

